# Low HPV16 E6 Seroprevalence in HNSCC: A Prospective Study in Brazil

**DOI:** 10.3390/jcm15093557

**Published:** 2026-05-06

**Authors:** Enes Buck Mutiua Cantala Xavier, Camila Batista Daniel, Priscila Marinho de Abreu, Isabella Bittencourt do Valle, Brena Ramos Athaydes, Frederico Firme Figueira, Agenor Sena, Evandro Duccini de Souza, Tim Waterboer, Sandra Ventorin von Zeidler

**Affiliations:** 1 Faculty of Health Sciences, Zambeze University, Beira 2300, Mozambique; enesbuckxavier@gmail.com; 2 Biotechnology Post-Graduation Program Rede Nordeste—RENORBIO, Health Sciences Center, Federal University of Espírito Santo, Vitória 29040-090, Espírito Santo, Brazil; 3 Biotechnology Post-Graduation Program, Health Sciences Center, Federal University of Espírito Santo, Vitória 29040-090, Espírito Santo, Brazil; batistad.camila@gmail.com (C.B.D.); priscilamarinho84@gmail.com (P.M.d.A.); isabellabdv@gmail.com (I.B.d.V.); brena.ramos@hotmail.com (B.R.A.); 4 Molecular Pathology Laboratory, Pathology Department, Health Sciences Center, Federal University of Espírito Santo, Vitória 29040-090, Espírito Santo, Brazil; frederico_firme@hotmail.com; 5 Head and Neck Surgery Division, Santa Rita de Cássia Hospital, Vitória 29043-260, Espírito Santo, Brazil; agenorsena@yahoo.com.br (A.S.); evandroduccini@gmail.com (E.D.d.S.); 6 Infections and Cancer Epidemiology, German Cancer Research Center (Deutsches Krebsforschungszentrum), 69120 Heidelberg, Germany; t.waterboer@dkfz-heidelberg.de

**Keywords:** squamous cell carcinoma, head and neck neoplasms, Papillomaviridae, human papillomavirus 16, seroepidemiologic studies, cohort studies

## Abstract

**Background/Objectives:** Head and neck squamous cell carcinoma (HNSCC) remains a global public health challenge with significant morbidity and mortality. Emerging epidemiological data indicate a rising global incidence of human papillomavirus (HPV)-related oropharyngeal squamous cell carcinoma (OPSCC). Serology for early HPV antigens has been highlighted as a relevant biomarker for HPV-associated OPSCC. This study aimed to determine the seroprevalence of HPV16 E6 antibodies in HNSCC patients in the state of Espírito Santo, Brazil. **Methods:** This is a prospective longitudinal cohort study in which 287 patients with HNSCC were enrolled, recruited from two oncology centers in Espírito Santo between 2011 and 2018, along with 68 cancer-free individuals. Serum samples were analyzed using the HPV16 E6 GST Capture ELISA assay. Statistical analysis was performed using SPSS. Binary logistic regression was employed to identify independent predictors of seropositivity. **Results:** The overall seroprevalence of HPV16 E6 antibodies was 7.3%. Seropositivity was observed in tumors of the oral cavity (6.2%) and oropharynx (13.3%). Patients with OPSCC demonstrated a significantly higher likelihood of seropositivity compared to those with tumors of the oral cavity, larynx, and hypopharynx (OR = 2.96, 95% CI: 1.21–7.28, *p* = 0.018). The highest frequency of HPV16 E6-positive cases occurred in tumors of the palatine tonsils (OR = 6.00; 95% CI: 1.58–22.89; *p* < 0.009). No seropositive cases were observed in hypopharyngeal or laryngeal tumors. Among patients with OPSCC and oral cavity squamous cell carcinoma (OCSCC), HPV16 E6 serostatus did not significantly correlate with sociodemographic, behavioral, or clinical tumor characteristics. **Conclusions:** Our findings reinforce the predilection of HPV-associated carcinogenesis for the oropharynx, more specifically in the palatine tonsils. In addition, this study highlights HPV16 E6 serology as a potential biomarker for HPV-driven OPSCC and underscores Brazil’s epidemiological heterogeneity, warranting standardized clinical validation.

## 1. Introduction

Head and neck squamous cell carcinoma (HNSCC) encompasses a group of epithelial malignancies arising from the oral cavity, pharynx, and larynx. This disease accounts for approximately 4% of the global cancer burden, with an estimated 771,000 new cases and 384,600 deaths annually [1]. In South America, HNSCC incidence and mortality rates vary considerably by country, with Brazil reporting the highest incidence, with over 28,000 new cases documented annually [2,3].

Historically, HNSCC has been strongly associated with tobacco and alcohol exposure, which remain the primary etiologic factors across multiple subsites. Nevertheless, over the past two decades, high-risk human papillomavirus (HR-HPV) infection has emerged as a distinct and significant risk factor for a specific subset of HNSCC, particularly those arising in the oropharynx [4,5,6,7,8,9]. According to worldwide cancer registry data (Cancer Incidence in Five Continents, years 1983 to 2002), the incidence of oropharyngeal squamous cell carcinoma (OPSCC) driven by HR-HPV has risen in several high-income nations [10,11,12]. In the United States, the proportion of HPV-associated OPSCC increased from 16.3% to 71% over the last four decades [13]. Similar upward trends have also been documented across Europe [14,15].

In contrast, the prevalence of HPV in OPSCC within Latin America appears less frequent and remains highly heterogeneous. In Brazil, HPV-associated OPSCC prevalence ranges from 4.1% to 31.9%, exhibiting substantial geographic variations [16,17,18,19,20]. These reported fluctuations across countries and regions likely reflect divergent sexual behaviors, tobacco exposure levels, diagnostic methodologies, and varying access to molecular testing [2,12,21,22,23].

HPV-associated OPSCC constitutes a biologically distinct subset of head and neck cancer, differing clinically, histologically and molecularly from smoking- and alcohol-driven HNSCC [24,25,26,27]. Notably, these tumors are frequently associated with improved clinical outcomes compared to HPV-independent OPSCC, when managed with equivalent therapeutic modalities [22,28,29,30,31]. Given these distinctions, accurately differentiating HPV-associated OPSCC from HPV-independent HNSCC is essential for epidemiological surveillance, precise prognostication, and the identification of patients who may benefit from optimized therapeutic strategies [32,33,34,35,36].

Serological markers of HPV exposure, particularly antibodies against viral oncoproteins such as E6, have emerged as robust biomarkers of HPV-driven carcinogenesis (sensitivity 83.1–97%; specificity 94.6–98%) and serve as a reliable tool for determining HPV status in clinical or epidemiological settings where tumor tissue is unavailable for conventional molecular testing [37,38].

Assessing anti-E6 seropositivity in HNSCC patients is instrumental in elucidating the burden of HPV-driven disease, as well as its regional profiles and epidemiological trends. This is particularly relevant in Brazil, where the reported prevalence of HPV-associated OPSCC remains comparatively low and high-quality data in this field are limited [16,39,40]. In this context, the present study aimed to determine the seroprevalence of HPV16 E6 antibodies among HNSCC patients in the state of Espírito Santo, Brazil, to better characterize the oncogenic contribution of HPV within this population.

## 2. Materials and Methods

### 2.1. Study Design and Sample Collection

This prospective longitudinal cohort study, conducted in Espírito Santo, southeastern Brazil, adhered to Declaration of Helsinki principles and Strengthening the Reporting of Observational Studies in Epidemiology (STROBE) guidelines [41]. Ethical approval was obtained from Cassiano Antônio de Moraes University Hospital, Vitória-ES, Brazil (Protocol No. 318/2011), and the National Research Ethics Commission (Protocol No. 681/2011). All participants provided written informed consent before enrollment.

Patients aged ≥18 years, of both sexes, with histologically confirmed, treatment-naïve head and neck squamous cell carcinoma (HNSCC) were recruited at diagnosis (2011–2018) from Head and Neck Surgery Divisions at Santa Rita de Cássia Hospital (AFECC) and Cassiano Antônio de Moraes University Hospital (HUCAM), both located in Vitória, Espírito Santo, Brazil. Of 303 recruited patients, 16 were excluded for missing ICD-O-3 codes (C31.0, C14.0, C12.9, C08.0, or undefined codes) or clinical data (e.g., smoking status, alcohol consumption, or tumor staging), yielding a convenience sample of 287 patients for final analysis (Supplementary Figure S1).

Serum samples were obtained from all individuals diagnosed with primary squamous cell carcinoma located in the oral cavity (C02-C06.2), oropharynx (C01; C01.9; C02.4; C05.1-C05.9; C09-C10.9), hypopharynx (C12, C12.1, and C13.8), and larynx (C32-C32.9), as well as unspecified subsites of oral cavity and oropharynx (C02.8, C02.9; C10.8, and C10.9). Clinical data, including primary tumor anatomical site and clinical stage at diagnosis, were retrieved from medical records or during medical consultations, according to the International Classification of Diseases for Oncology, 3rd edition [42] and the TNM Classification System, 7th edition [43], respectively. 

Additionally, 68 serum samples were collected from individuals with no prior history of cancer admitted to HUCAM, matched by sex, self-reported skin color, and age (±5 years) to establish the laboratory’s seropositivity threshold (cut-off) for our population.

### 2.2. Measurement of HPV16 E6 Antibodies Using GST Capture ELISA

Serum levels of HPV16 E6 antibodies were quantified using a glutathione S-transferase (GST) capture ELISA developed at the German Cancer Research Center (DKFZ), following previously established protocols [44]. Briefly, the serum samples were diluted to 1:100 in a buffer containing casein and lysate from *E. coli* overexpressing GST. These diluted samples were incubated for 1 hour at room temperature before being transferred to ELISA plates blocked with casein and coated with affinity-purified GST-HPV16 E6 lysate. The plates were then incubated for an additional hour at room temperature.

Following three washes with phosphate-buffered saline containing 0.05% Tween (PBS-T), 100 µL of anti-human IgG-peroxidase-conjugated secondary antibody (Dianova 109-035-064, diluted 1:20,000) was added to each well. The plates were incubated at room temperature for 1 h, protected from direct light. After a subsequent three-wash cycle with PBS-T, 100 µL of tetramethylbenzidine (TMB) was added to each well and incubated for 8 min in the dark. The enzymatic reaction was stopped by adding 50 µL of 1M sulfuric acid per well. The optical density (OD) of the samples was measured at a wavelength of 450 nm using a Synergy^®^ H1 microplate reader (Thermo Fisher Scientific Inc., Waltham, MA, USA).

Internal quality control was strictly maintained on each plate using background controls (buffer only) and confirmed HPV-seronegative sera. To ensure inter-assay consistency and longitudinal standardization, a pooled HPV16 E6-seropositive reference serum (OPC-pool) was analyzed in serial 1:2 dilutions (1:50–1:6400) [45].

### 2.3. Multiplex Serology

Multiplex serologic testing was performed at the German Cancer Research Center (Heidelberg, Germany). Antigens were affinity-purified, bacterially expressed fusion proteins with N-terminal Glutathione S-transferase. Samples were analyzed for antibodies against the HPV16 E6 protein. Antibody levels were quantified at 1:100 serum dilution as median fluorescence intensity (MFI) and dichotomized as positive or negative based on previously defined cut points [46].

### 2.4. Data Analysis

HPV16 E6 seropositivity was determined based on OD readings obtained at 450 nm. Cut-off for seropositivity was defined as the mean OD of sera from individuals with no prior history of cancer plus three standard deviations (X ± 3*SD), following the methodology described by Crowther and Muller [47,48]. To ensure a robust threshold, an iterative outlier exclusion process was employed: serum samples with absorbance values exceeding the initial calculated cut-off were considered outliers and excluded, after which the mean and standard deviation were recalculated using the remaining samples [47]. The mean OD of this group was 0.061 (SD = 0.033), resulting in a definite cut-off value of 0.159. Samples with an OD equal to or greater than this value were classified as seropositive for anti-HPV16 E6 antibodies.

Descriptive statistics were expressed as measures of central tendency and dispersion for continuous variables, and as absolute and relative frequencies for categorical variables. HPV-attributable fractions (AFs) were calculated across sociodemographic characteristics, risk factors, and tumor sites. To identify independent predictors of HPV16 E6 seropositivity, a binary logistic regression model (using the ENTER method) was employed, with results reported as odds ratios (ORs) and 95% confidence intervals (CIs). The proportion of HPV-OPC cases seropositive for HPV16 antibodies (sensitivity) and the proportion of HPV-negative OPCs seronegative for HPV16 antibodies (specificity) were calculated for E6 HPV16 protein using the multiplex serology as the reference standard; confidence intervals (CIs) were estimated using an exact binomial method. All analyses were performed using SPSS version 23 (IBM Corp., Armonk, NY, USA), and a *p*-value < 0.05 was considered statistically significant.

## 3. Results

### 3.1. Characteristics of the Study Population

The HNSCC study population comprised 287 individuals, predominantly male (84.3%), aged ≥45 years (90.5%), and self-identified as mixed race (48.0%). Alcohol consumption was reported by 86.1% of patients, including 48.9% current drinkers and 37.2% former drinkers. Similarly, a history of tobacco use was reported by 81.9% of the population, of whom 53.9% were current smokers and 28.0% former smokers. Notably, the highest proportion of current smokers was observed among patients with hypopharyngeal tumors (71.4%).

The oral cavity was the most frequent primary tumor site, accounting for 56.4% of HNSCC cases, followed by the oropharynx (28.9%), larynx (9.8%), and hypopharynx (4.9%). Most patients presented with locally advanced disease at diagnosis, with 63.2% exhibiting T3/T4 tumor size and 69.0% classified as clinical stages III and IV. Comprehensive sociodemographic, risk exposure, and clinicopathological data are summarized in Table 1.

### 3.2. HPV16 E6 Seroprevalence in HNSCC Cases

**Figure 1:**
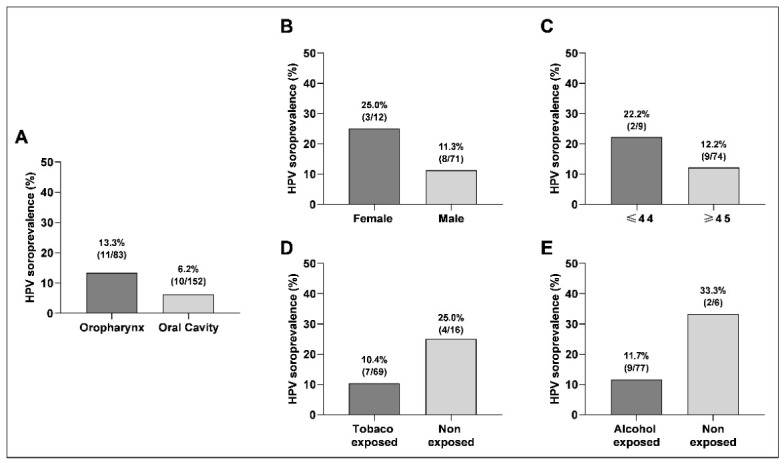
Prevalence of HPV E6 seropositivity according to anatomical site and demographic and behavioral characteristics. (**A**) Prevalence in oral cavity and oropharyngeal squamous cell carcinoma (OPSCC). (**B**–**E**) Prevalence among patients with OPSCC, according to (**B**) sex, (**C**) age group, (**D**) history of tobacco exposure, (**E**) history of alcohol exposure.

HPV16 E6 seropositivity was detected in 13.3% (*n* = 11/83) of OPSCC patients and 6.2% (*n* = 10/152) of those with oral cavity squamous cell carcinoma (OCSCC). This corresponds to an overall seroprevalence of 7.3% (*n* = 21/287) across the entire HNSCC cohort (Figure 1A).

Patients with OPSCC were significantly more likely to be HPV16 E6-seropositive compared to those with tumors at other anatomical sites (OR = 2.964; 95% CI: 1.207–7.276; *p* = 0.018) (Table 2). Furthermore, the highest prevalence of HPV16 E6-positive cases was identified in the palatine tonsils (OR = 6.000; 95% CI: 1.573–22.889; *p* = 0.009). These data indicate that HPV16 E6-seropositive individuals have a six-fold higher likelihood of presenting with a malignancy in this specific anatomical subsite compared to other subsites within the oropharynx (Table 2).

Although the OPSCC cohort was characterized by a predominance of males, individuals aged ≥45 years, and a history of tobacco and alcohol consumption, no significant associations were identified between these sociodemographic or behavioral variables and HPV16 E6 serological status (Table 3). However, despite the limitations of the current sample size, certain descriptive trends emerged. A higher relative prevalence of HPV16 E6 antibodies was observed among females (25.0%), individuals aged ≤44 years (22.2%), never-drinkers (33.3%), and never-smokers (25.0%) (Figure 1B–E), suggesting a potentially distinct profile for HPV-driven cases within the oropharynx.

HPV16 E6 GST-ELISA results were compared to multiplex serology (reference standard), yielding a sensitivity of 33.3% and specificity of 98.2% (Supplementary Table S1).

## 4. Discussion

This is the first study to report the prevalence of HPV16 E6 antibodies among HNSCC patients in the state of Espírito Santo, Brazil. Overall, HPV16 E6 seropositivity was detected in 7.3% of cases, with significantly higher frequency observed in oropharyngeal cancers (13.3%), particularly in tumors located in the palatine tonsils (OR = 6.000). These findings highlight the established anatomical predilection of HPV-associated carcinogenesis for the oropharynx, consistent with previous reports demonstrating the disproportionate burden of HPV in this subsite compared to other head and neck regions [7,8,49,50].

To date, only a limited number of studies have evaluated HPV16 E6 seroprevalence in HNSCC within the Brazilian context [16,39]. Overall, the prevalence of HPV16-related OPSCC in Brazil remains substantially lower (ranging from 4.1% to 31.9%) than rates reported in North America, Europe, Asia, and other Latin American countries, where frequencies range from 22% to 84% [12,22,23,51]. Notably, the HPV16 E6 seroprevalence observed in our OPSCC cohort (13.3%) was slightly higher than the rates previously reported by Sichero et al. (10.6%) and Lopez et al. (10.7%) in other Brazilian states [16,39]. These differences may underlie distinct approaches in defining seropositivity thresholds. Furthermore, the temporal and geographical scope of the cohorts must be considered. López et al., 2014 analyzed a large multicenter cohort recruited between 1998 and 2008 across several Brazilian cities in Southeast and Central-West [16]. Conversely, Sichero et al., 2024 focused on a more recent series (2015–2019) from a single institution in São Paulo [39]. By providing the first serological data from the state of Espírito Santo, our findings highlight potential regional variations in the HPV-attributable fraction within Brazil’s diverse landscape.

Nevertheless, the seroprevalence of 13.3% observed in our OPSCC cohort is lower than the prevalence reported in studies from other Brazilian regions, where HPV16 tumor detection was performed using polymerase chain reaction (PCR) and p16 immunohistochemistry, with rates ranging from 15.5% to 31.9% [18,19,20,52,53]. This discrepancy likely reflects methodological differences between direct tumor-based HPV detection and serological assessment. Such variation often suggests an overestimation of HPV-attributable fractions when tumors are classified as HPV-related based solely on p16 or HPV DNA PCR, particularly when these markers are utilized in isolation [37]. Comparing these markers with serological detection requires a nuanced interpretation of sensitivity and specificity. E6 serology may exhibit reduced sensitivity when compared to the “true” HPV-driven status, such as those defined by the concomitant presence of viral DNA and E6 mRNA. However, when compared to other biomarkers, serology can demonstrate superior specificity and, in certain contexts, higher sensitivity for identifying biologically active infections [37,38,39,46]. In our study, the low sensitivity (33.3%) should be interpreted with caution, as it reflects the limited number of positive cases in this randomly selected subset. The small sample size substantially limits the precision and interpretability of this estimate. Notably, the high specificity (98.2%) confirms the assay’s reliability for identifying true negatives. However, the paucity of positive samples precluded a more comprehensive evaluation of assay performance.

In addition, variations in HPV detection methods, diagnostic criteria, and the sociodemographic composition of study populations may contribute to the heterogeneity observed across Brazilian studies. Furthermore, the temporal increase in HPV-positive cases described by Carvalho et al. and Pires et al. [18,19] suggests a dynamic epidemiological shift that may not manifest uniformly in all regions of the country. Such disparities may be attributed to regional variations in HPV transmission patterns, sex distribution, ethnicity, and tobacco and alcohol consumption, representing a complex interplay of socioeconomic, environmental, and biological determinants [22,54]. Furthermore, it is important to acknowledge that, in populations with a high burden of other HPV-related malignancies, such as anogenital cancers, the detection of anti-E6 antibodies may reflect extra-oropharyngeal infections rather than a head and neck primary tumor. Therefore, while serological biomarkers are highly sensitive for detecting HPV-attributable fraction tumors, they should be utilized considering these aspects.

Our results reinforce the significant association between HPV16 E6 seropositivity and OPSCC, likely influenced by the unique anatomical and immunological characteristics of the oropharynx that facilitate viral persistence [26,51]. The concentration of HPV16 E6-positive cases specifically within the palatine tonsils confirms the strong anatomical predilection of HPV for the oropharynx, particularly for tonsillar crypt epithelium. The magnitude of this effect, characterized by a six-fold increase in the likelihood of malignancy at this subsite among seropositive individuals, aligns with established national and international evidence identifying the palatine tonsils as a primary site of HPV-associated carcinogenesis [49].

Furthermore, our findings indicated a higher seroprevalence among females, younger individuals, and those with no exposure to alcohol or tobacco within our OPSCC cohort. This pattern likely reflects the emerging epidemiological profile of HPV-driven OPSCC, which frequently presents in younger patients and in those lacking traditional synergistic risk factors such as tobacco and alcohol consumption [55].

The detection of antibodies against HPV oncoproteins, particularly E6, represents a promising strategy for the epidemiological surveillance of virus-associated malignancies. Anti-HPV16 E6 serology facilitates the identification of prior oncogenic exposure, estimation of the HPV-attributable disease burden, monitoring of temporal trends, and evaluating preventive measures, thereby broadening our understanding of the epidemiological dynamics of these cancers. Multiple studies have demonstrated the accuracy of HPV16 serology, particularly E6 serology, in detecting HPV-associated HNSCC, with pooled sensitivity and specificity of 83.1% and 94.6%, respectively [16,37,39,54], positioning it as a clinically relevant marker of active oncogenesis. However, the gold standard remains the combined use of two markers (p16 immunohistochemistry plus HPV16 DNA detection or in situ hybridization). This dual approach minimizes false positives from p16 alone and confirms transcriptionally active HPV-driven oncogenesis with high accuracy, achieving sensitivity above 90% [37]. Furthermore, E6 seropositivity has brought new contributions, such as being detected several years before the onset of clinical symptoms, introducing new opportunities for early detection and preventive strategies, even in regions with modest seroprevalence [37,46].

Finally, HPV status is a well-established prognostic indicator in OPSCC, as HPV-associated tumors typically exhibit superior survival rates and enhanced therapeutic responses compared to HPV-independent disease. Several studies demonstrate that HPV-16 E6 seropositivity at the time of diagnosis is a powerful diagnostic tool and independent predictor of improved overall survival [16,38,39,46]. Our findings reinforce the utility of assessing seroprevalence via anti-HPV16 E6 antibodies to identify patients with these biologically favorable, virus-driven tumors during the initial clinical workup. While these antibodies are long-lasting and not site-specific, limiting their application for post-treatment monitoring or recurrence detection, their presence at diagnosis serves as a potential tool for pretreatment risk stratification [16,27,46].

A significant strength of this study is the large cohort of HNSCC patients analyzed, particularly those with OPSCC. However, a limitation lies in the “unspecified” classification assigned to certain tumors of the oral cavity (e.g., C02.8, C02.9) and oropharynx (e.g., C10.8, C10.9). In our cohort, the largest E6-positive subgroup within the oral cavity, coded as C02.9, may have been misclassified and could, in fact, represent oropharyngeal tumors. Such diagnostic ambiguity presents a challenge for precise tumor staging, epidemiological analyses, and clinical management, requiring urgent attention from physicians and other health professionals. This lack of anatomical specificity can hinder the identification of patient subgroups with similar prognoses and therapeutic responses, potentially affecting staging and individualized treatment decisions. Furthermore, the absence of concurrent tissue-based analysis (e.g., p16 IHC or HPV DNA PCR) limits the direct correlation between serological findings and intratumoral HPV status. Furthermore, while HPV16 E6 seropositivity is a highly specific marker for HPV-driven malignancy, the absence of integrated molecular and immunohistochemical analyses (such as HPV DNA and p16 IHC) for all cases limited a full characterization of the tumor’s viral status, highlighting the need for future longitudinal studies that integrate molecular and epidemiological frameworks.

Despite these limitations, our study provides critical baseline data on HPV16 E6 seroprevalence in HNSCC in an underrepresented Brazilian cohort within the state of Espírito Santo, contributing valuable data to the still-limited set of seroepidemiologic studies in Brazil. By demonstrating a site-specific association, a substantial effect on the palatine tonsils, and a distinct demographic profile suggestive of HPV-related disease, our findings reinforce the biological and epidemiological relevance of HPV16 E6 as a marker of virus-related OPSCC. In a nation marked by vast regional heterogeneity and evolving epidemiological trends, these data underscore the importance of integrating serological biomarkers into multi-center surveillance structures to refine burden estimates, improve risk stratification, and support evidence-based clinical and public health strategies tailored to the Brazilian context.

## 5. Conclusions

Collectively, our findings reinforce the anatomical specificity of HPV-associated carcinogenesis in HNSCC and highlight the potential of HPV16 E6 serology as a complementary tool for stratifying patients according to tumor site. Future research is warranted to further validate the detection of HPV16 E6 by ELISA assay as a biomarker of active HPV infection, and to establish standardized parameters for its clinical application in early detection and prognostic assessment.

## Figures and Tables

**Table 1:** Baseline characteristics of HNSCC patients: sociodemographic, risk exposure, and tumor features (*n* = 287).

Variables		Oral Cavity	Oropharynx	Larynx	Hypopharynx	Total
		*n* (%)	*n* (%)	*n* (%)	*n* (%)	*n* (%)
Sex						
	Male	131 (80.7)	71 (85.5)	26 (92.9)	14 (100)	242 (84.3)
	Female	31 (19.3)	12 (14.5)	2 (7.1)	-	45 (15.7)
						
Age (years)						
	<44	16 (10.0)	9 (10.8)	1 (3.6)	1 (7.1)	27 (9.5)
	≥45	144 (90.0)	74 (89.2)	27 (96.4)	13 (92.9)	258 (90.5)
						
Skin color						
	Black	23 (14.6)	8 (9.6)	1 (3.7)	3 (21.4)	35 (12.5)
	White	59 (37.6)	35 (42.2)	11 (40.7)	5 (35.7)	110 (39.1)
	Mixed race	75 (47.8)	39 (47.0)	15 (55.6)	6 (42.9)	135 (48.0)
	Indigenous	-	1 (1.2)	-	-	1 (0.4)
						
Alcohol use						
	Current	78 (49.7)	40 (48.2)	14 (50.0)	6 (42.9)	138 (48.9)
	Former	49 (31.2)	37 (44.6)	12 (42.9)	7 (50.0)	105 (37.2)
	None	30 (19.1)	6 (7.2)	2 (7.1)	1 (7.1)	39 (13.9)
						
Tobacco use						
	Current	91 (58.0)	39 (47.0)	12 (42.9)	10 (71.4)	152 (53.9)
	Former	36 (22.9)	28 (33.7)	12 (42.9)	4 (28.4)	79 (28.0)
	None	30 (19.1)	16 (19.3)	4 (14.3)	-	51 (18.1)
						
Tumor size						
	T1/T1	63 (40.1)	22 (27.2)	16 (57.1)	2 (14.3)	103 (36.8)
	T3/T4	94 (59.9)	59 (72.8)	12 (42.9)	12 (85.7)	177 (63.2)
						
Node metastasis						
	N0	91 (58.0)	35 (42.7)	22 (78.6)	4 (28.6)	152 (54.1)
	N+	66 (42.0)	47 (57.3)	6 (21.4)	10 (71.4)	129 (45.9)
						
Clinical stage						
	I/II	53 (33.8)	18 (22.0)	15 (53.6)	1 (7.1)	87 (31.0)
	III/IV	104 (66.2)	64 (78.0)	13 (46.4)	13 (92.9)	194 (69.0)
						
Total		162	83	28	14	287

Missing data: age (*n* = 2); color (*n* = 6); alcohol consumption (*n* = 5); tobacco consumption (*n* = 5); tumor size (*n* = 7); lymph node metastasis (*n* = 6); distant metastasis (*n* = 6); clinical staging (*n* = 6); vital state (*n* = 8).

**Table 2:** Association between HPV16 E6 seroprevalence and the primary anatomical HNSCC site (*n* = 287).

Primary Tumor Site		HPV16 E6 Serology		OR	95% CI	*p*-Value
		Positive	Negative			
		*n* (%)	*n* (%)			
Oropharynx		11 (52.4)	72 (27.1)	2.964	(1.207–7.276)	0.018
	Base of tongue ^1^	2 (09.5)	31 (13.8)	0.294	(0.59–1.458)	0.134
	Soft palate ^2^	1 (04.8)	10 (04.5)	0.620	(0.071–5.384)	0.665
	Palatine tonsils ^3^	6 (28.6)	12 (05.4)	6.000	(1.573–22.889)	0.009
	Oropharynx wall ^4,a^	0 (00.0)	05 (02.2)	-	-	-
	Oropharynx unspecified ^5^	2 (09.5)	14 (06.3)	0.921	(0.179–4.744)	0.921
						
Oral Cavity		10 (47.6)	152 (57.1)	0.682	(0.280–1.66)	0.399
	Oral tongue ^6^	2 (09.5)	61 (27.2)	0.373	(0.077–1.816)	0.222
	Tongue unspecified ^7^	3 (14.3)	15 (06.7)	3.914	(0.915–16.750)	0.066
	Gum ^8,a^	0 (00.0)	09 (04.0)	-	-	-
	Floor of mouth ^9^	2 (09.5)	39 (17.4)	0.724	(0.147–3.558)	0.691
	Hard palate ^10,a^	0 (00.0)	03 (01.3)	-	-	-
	Oral mucosa ^11^	1 (04.8)	16 (07.1)	0.944	(0.112–7.947)	0.958
	Retromolar area ^12^	2 (09.5)	09 (04.0)	3.972	(0.733–21.518)	0.110
						
Larynx ^b^		-	28 (10.5)	<0.001	-	0.998
Hypopharynx ^b^		-	14 (5.3)	<0.001	-	0.998

OR: Odds ratio. Reference category of dependent variable: Primary tumor site—Oral cavity site: yes; Primary tumor site—Oropharynx site: no. Subsites: Oral tongue, Tongue unspecified, Floor of mouth, Oral mucosa, Retromolar area, Base of tongue, Soft palate, Palatine tonsils, and oropharynx unspecified: no. a—There are no statistical findings due to the absence of HPV-positive cases. b—There are no HPV-positive cases. ^1^ C01, C01.9, C02.4/ ^2^ C05.1, C05.2/ ^3^ C09-C09.9/ ^4^ C09-C09.9/ ^5^ C10.8, C10.9/ ^6^ C02-C02.3/ ^7^ C02.8, C02.9/ ^8^ C03, C03.1, C03.9/ ^9^ C04, C04.1, C04.8, C04.9/ ^10^ C05.0/ ^11^ C06-C06.9/ ^12^ C06.2.

**Table 3:** Association between HPV16 E6 seroprevalence in oral cavity and oropharynx squamous cell carcinoma and demographic characteristics, risk exposure, and tumor features (*n* = 245).

Variables	OPSCC					OCSCC				
	HPV +	HPV −	OR	95% CI	*p*	HPV +	HPV −	OR	95% CI	*p*
	*n* (%)	*n* (%)				*n* (%)	*n* (%)			
Sex										
Female	3 (27.3)	9 (12.5)	2.51	(0.55–11.40)	0.47	1 (10.0)	30 (19.7)	1.29	(0.41–4.03)	0.66
Male	8 (72.7)	63 (87.5)	1	-		9 (90.0)	122 (80.3)	1	-	
Age (years)										
≤44	2 (18.2)	7 (9.7)	0.52	(0.09–3.01)	0.23	2 (22.2)	14 (9.3)	2.63	(0.81–8.52)	0.10
≥45	9 (81.8)	65 (90.3)	1	-		7 (77.8)	137 (90.7)	1	-	
Skin color										
Black	1 (9.1)	7 (9.7)	0.92	(0.10–8.36)	0.94	0 (0.0)	23 (15.5)	2.84	(0.36–21.95)	0.31
White	6 (54.5)	29 (40.3)	1.77	(0.49–6.37)	0.37	2 (22.2)	57 (38.5)	0.96	(0.38–2.43)	0.93
Mixed race	4 (36.4)	35 (48.6)	1.65	(0.44–6.15)	0.45	7 (77.8)	68 (45.9)	0.75	(0.29–1.84)	0.51
Indigenous	-	1 (1.4)	-	-	-	-	-	-	-	-
Alcohol consumption										
Current	4 (36.3)	36 (50.0)	1.75	(0.47–6.50)	0.40	6 (66.7)	72 (48.6)	0.95	(0.38–2.37)	0.92
Former	5 (45.5)	32 (44.4)	0.96	(0.26–3.43)	0.95	1 (11.1)	48 (32.4)	1 41	(0.52–3.80)	0.48
None	2 (18.2)	4 (5.6)	0.26	(0.42–1.65)	0.15	2 (22.2)	28 (18.9)	0.61	(0.19–1.95)	0.41
Tobacco use										
Current	4 (36.4)	35 (48.6)	1.65	(0.44–6.15)	0.45	4 (44.4)	87 (58.8)	1 83	(0.72–4.62)	0.20
Former	3 (27.3)	25 (34.7)	1.41	(0.34–5.82)	0.62	4 (44.4)	32 (21.6)	0.71	(0.27–1.86)	0.49
None	4 (36.4)	12 (16.7)	0.35	(0.88–1.38)	0.13	1 (11.1)	29 (19.6)	0.62	(0.21–1.79)	0.38
Tumor size										
T1/T2	3 (27.3)	19 (27.1)	0.99	(0.23–4.14)	0.99	3 (33.3)	60 (40.5)	1.38	(0.51–3.73)	0.51
T3/T4	8 (72.7)	51 (72.9)	1	-		6 (66.7)	88 (59.5)	1	-	
Lymph node metastasis										
N0	3 (27.3)	32 (45.1)	2.18	(0.53–8.93)	0.27	6 (66.7)	85 (57.4)	1.48	(0.59–3.69)	0.40
N+	8 (72.7)	39 (54.9)	1	-		3 (33.3)	63 (42.6)	1	-	
Clinical staging										
I/II	3 (27.3)	15 (21.1)	0.71	(0.16–3.02)	0.64	2 (22.2)	51 (34.5)	1.37	(0.48–3.90)	0.55
III/IV	8 (72.7)	56 (78.9)	1	-		7 (77.8)	97 (65.5)		-	

Missing data: age (*n* = 2); color (*n* = 6); alcohol consumption (*n* = 5); tobacco use (*n* = 5); tumor size (*n* = 7); lymph node metastasis (*n* = 4); distant metastasis (*n* = 4); clinical staging (*n* = 6).

## Data Availability

Due to ethical considerations, data cannot be made publicly available. Anonymized data will be made available by the authors upon reasonable request.

## Supplementary Materials

The following supporting information can be downloaded at: https://www.mdpi.com/article/10.3390/jcm15093557/s1, Figure S1: Diagram illustrating the patient selection process; Table S1: Sensitivity and specificity of serum HPV antibodies relative to the reference detection method.

## Abbreviations

The following abbreviations are used in this manuscript:

HNSCC	Head and Neck Squamous Cell Carcinoma
OPSCC	Oropharyngeal Squamous Cell Carcinoma
OCSCC	Oral Cavity Squamous Cell Carcinoma
HPV	Human Papillomavirus
HR-HPV	High-risk human papillomavirus
OR	Odds Ratio
CI	Confidence interval
SD	Standard Deviation
HUCAM	Hospital Universitário Cassiano Antônio de Moraes
AFECC	Women’s Association for Education and Fight Against Cancer
ELISA	Enzyme-Linked Immunosorbent Assay
IHC	Immunohistochemistry
