# Supplementary material for: Low HPV16 E6 Seroprevalence in HNSCC: A Prospective Study in Brazil
Source: J Clin Med. 2026 May 6;15(9):3557. doi: 10.3390/jcm15093557 (PMC13164344; doi:10.3390/jcm15093557)
Supplement: Supplementary file 1 [file jcm-15-03557-s001.zip › jcm-4226042-supplementary.pdf]

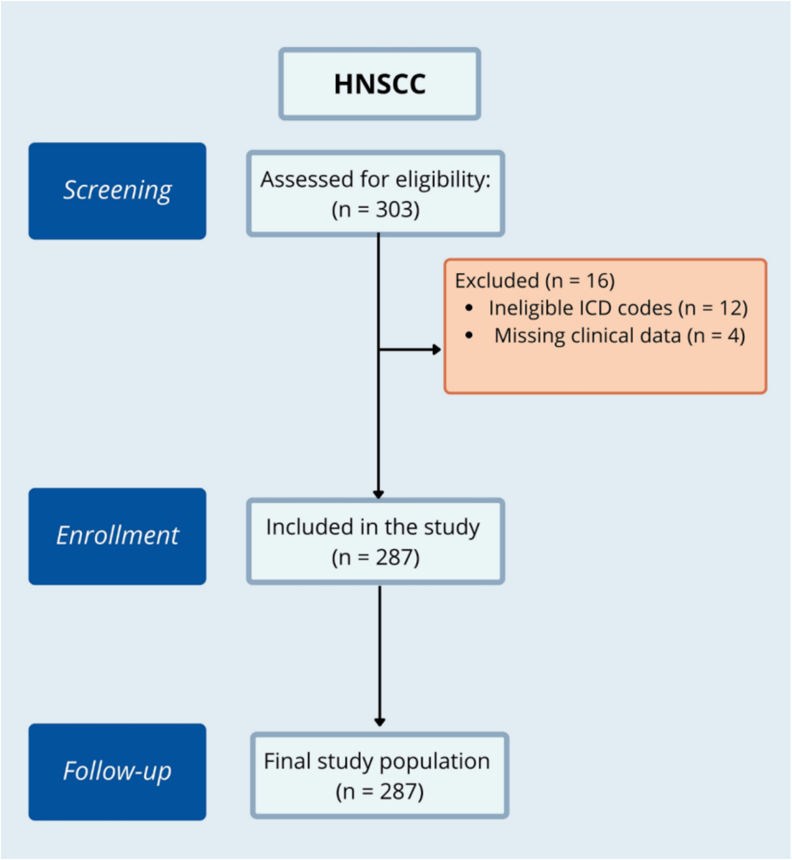

Supplementary Figure S1. Diagram illustrating the patient selection process.

Abbreviations: HNSCC: Head and neck squamous cell carcinoma. ICD: International Classification of Diseases.

Supplementary Table S1. Sensitivity and specificity of serum HPV antibodies relative to the reference detection method.

	HR-HPV Reference by LUMINEX				
**Serostatus**	**HPV +** (n = 3)	**HPV -** (n = 112)	**Sensitivity (95% CI)**	**Specificity (95% CI)**	***p*-Value**
HPV Seropositive	1	2	33.3 (1.71 – 88.15)	98.2 (93.7 -99.7)	<0.001*
HPV Seronegative	2	110			

Abbreviations: HR-HPV: High-risk human papillomavirus.
* Fisher's exact test.
